# Metabolically healthy obesity: What is the role of sedentary behaviour?^☆^

**DOI:** 10.1016/j.ypmed.2014.01.028

**Published:** 2014-05

**Authors:** Joshua A. Bell, Mika Kivimaki, G. David Batty, Mark Hamer

**Affiliations:** Department of Epidemiology & Public Health, University College London, 1-19 Torrington Place, London WC1E 6BT, UK

**Keywords:** Sedentary behaviour, Television viewing, Obesity, Metabolic health

## Abstract

**Objective:**

The role of sedentary behaviour in metabolically healthy obesity is unknown. We examined cross-sectional differences in television viewing time across metabolic and obesity phenotypes, hypothesizing that healthy obese individuals spend less time viewing television than their unhealthy counterparts.

**Methods:**

A nationally representative sample of 4931 older adults in England (mean age 65.1; SD = 8.9 years) was drawn from the 2008/9 wave of the English Longitudinal Study of Ageing. Average weekly television viewing time was derived from two questions about weekday and weekend viewing. Obesity was defined as body mass index ≥ 30 kg/m^2^, and metabolically healthy as having < 2 metabolic abnormalities (low HDL-cholesterol, high triglycerides, high blood pressure, hyperglycaemia, high inflammation).

**Results:**

After adjusting for covariates including chronic illness, functional limitations and physical activity, mean weekly viewing times were 4.7 (95% confidence interval 2.9, 6.5), 5.8 (2.5, 9.0) and 7.8 (5.7, 9.8) h higher in unhealthy non-obese, healthy obese, and unhealthy obese groups respectively, compared to the healthy non-obese group (p for heterogeneity < 0.001).

**Conclusions:**

A common type of leisure-time sedentary behaviour varies across metabolic and obesity phenotypes. However, healthy obesity is not explained through differences in leisure-time sedentary behaviour.

## Introduction

The growing recognition of a ‘metabolically healthy’ obese phenotype has fuelled efforts to identify its behavioural determinants. While recent cross-sectional evidence supports the role of physical activity (Wildman et al., 2008) and cardiorespiratory fitness (Ortega et al., 2013), sedentary behaviour has been associated with adverse levels of metabolic risk factors including blood pressure, glucose, and lipids, independent of engagement in moderate-to-vigorous intensity physical activity (Gardiner et al., 2011; Pereira et al., 2012). Sedentary behaviour is thought to represent a distinct state of muscle inactivity that may independently influence disease risk through a variety of underlying molecular mechanisms, including lipoprotein lipase pathways (Hamilton et al., 2007) and the expression of various genes linked to inflammatory responses (Latouche et al., 2013). Lower levels of sedentary behaviour may therefore help explain why some obese individuals are able to maintain metabolic health. As research has found associations between sitting and metabolic risk to be most pronounced when using television viewing as an indicator (Pereira et al., 2012; Stamatakis et al., 2012), we assessed differences in television viewing time across metabolic and obesity phenotypes, and hypothesized that metabolically healthy obese individuals would spend less time viewing television than their metabolically unhealthy counterparts.

## Methods

Self-reported television viewing time and objectively measured obesity phenotype status were collected during wave 4 (2008/9) of the English Longitudinal Study of Ageing (ELSA): an on-going, nationally representative, prospective cohort study of adults aged 50 years and over living in private households in England (Steptoe et al., 2012). Participants gave full-informed written consent. Ethical approval was obtained from the London Multi-Centre Research Ethics Committee.

Average weekly television viewing time was derived from two questions about weekday and weekend viewing: (hours per weekday ∗ 5 + total hours per weekend). Obesity was defined as body mass index ≥ 30 kg/m^2^. Metabolically healthy was defined as having < 2 of the following abnormalities: HDL-cholesterol < 1.03 mmol/L for men and < 1.29 mmol/L for women; triglycerides ≥ 1.7 mmol/L; blood pressure ≥ 130/85 mm Hg or taking anti-hypertension medication or doctor diagnosed hypertension; CRP inflammatory marker ≥ 3 mg/L; HbA1c ≥ 6% (International Federation of Clinical Chemistry HbA1c ≥ 42 mmol/mol) or taking diabetic medication or doctor diagnosed diabetes, based on comprehensive criteria (Wildman et al., 2008).

General linear models examined cross-sectional differences in television viewing time in relation to 4 metabolic health/obesity statuses: ‘metabolically healthy non-obese’ (reference group), ‘metabolically unhealthy non-obese’, ‘metabolically healthy obese’, and ‘metabolically unhealthy obese’. The first model adjusted for age and sex. The second model further adjusted for marital status, occupational class, self-reported presence of any long-standing illness which limits activities, limitations in basic and instrumental activities of daily living, depressive symptoms (based on 8-item Centre of Epidemiological Studies Depression Scale), and health behaviours including smoking status, frequency of alcohol consumption, and frequency of moderate–vigorous intensity physical activity. Analyses were performed using SPSS 21 with p < 0.05 signifying statistical significance.

## Results

The analytic sample comprised 2683 women and 2248 men, aged 65.1 (SD = 8.9) years (98% White British). Mean television viewing time for the entire sample was 36.6 (SD = 27.7) h/week. Adjusting for age and sex, mean viewing times were 31.4 (95% confidence interval 30.1, 32.6) h/week, 38.0 (36.6, 39.3) h/week, 38.8 (35.7, 41.9) h/week and 42.0 (40.4, 43.6) h/week for healthy non-obese, unhealthy non-obese, healthy obese, and unhealthy obese groups respectively (Supplementary Table 1).

The analytic sample comprised 2683 women and 2248 men, aged 65.1 (SD = 8.9) years (98% White British). Mean television viewing time for the entire sample was 36.6 (SD = 27.7) h/week. Adjusting for age and sex, mean viewing times were 31.4 (95% confidence interval 30.1, 32.6) h/week, 38.0 (36.6, 39.3) h/week, 38.8 (35.7, 41.9) h/week and 42.0 (40.4, 43.6) h/week for healthy non-obese, unhealthy non-obese, healthy obese, and unhealthy obese groups respectively (Supplementary Table 1).

Associations persisted after adjusting for socioeconomic factors, physical and mental health status, functional limitations, and health behaviours including moderate–vigorous intensity physical activity. Significant heterogeneity in television viewing time was observed across phenotypes (p < 0.001), with longer weekly viewing time associated with less favourable metabolic and obesity status. Compared with the healthy non-obese, excess television viewing time was 4.7 (2.9, 6.5) h/week, 5.8 (2.5, 9.0) h/week, and 7.8 (5.7, 9.8) h/week for unhealthy non-obese, healthy obese, and unhealthy obese groups respectively (Table 1). Pairwise comparisons and overlapping confidence intervals indicated that healthy non-obese adults viewed significantly less television per week than unhealthy non-obese adults (p < 0.001), while differences in television viewing time between healthy and unhealthy obese groups were not statistically significant (p = 0.252).

## Discussion

The role of physical activity and cardiorespiratory fitness in contributing to metabolically healthy obesity has been explored (Ortega et al., 2013; Wildman et al., 2008), but whether sedentary behaviour helps explain differences in metabolic health within the obese population has not been previously investigated. Our results suggest that levels of sedentary behaviour, as indicated by self-reported television viewing, vary across metabolic and obesity phenotypes; however healthy obese adults did not demonstrate significantly different television viewing time than their unhealthy counterparts after adjusting for socioeconomic, health, and behavioural covariates including physical activity. Significant differences in television viewing time between metabolically healthy and unhealthy non-obese groups were observed.

Television viewing was utilised here as the only marker of sedentary behaviour as past research has found associations between sitting and metabolic risk to be most pronounced in this context. Indeed, one study observed associations when sitting while viewing television but not while working (Pereira et al., 2012), while another observed associations during television viewing but not during other sedentary leisure activities (Stamatakis et al., 2011). The proportion of obese individuals who are metabolically healthy tends to decrease with increasing age (Wildman et al., 2008), and thus associations observed in present analyses may be underestimated for the obese population as a whole. Indeed, less than one quarter (20.9%) of our sample of obese older adults was considered metabolically healthy, while this proportion is nearly one-third considering all adults collectively when using similar criteria (Wildman et al., 2008). Results may also be complicated in older populations since lower body mass index in older people often relates to prevalent chronic disease (Mazza et al., 2006). Older adults who have retired may also spend a larger proportion of their day viewing television than younger adults. Future studies should examine associations in other age groups and across different domains of leisure and occupational sitting.

While this study accounted for a range of covariates relevant to older adults including chronic illness and functional limitations, snacking behaviour was not considered, although it is known to occur while viewing television (Gore et al., 2003). Previous work has shown associations between television viewing and metabolic abnormalities to persist after controlling for frequency of unhealthy food consumption (Stamatakis et al., 2011), but this behaviour may indeed confound associations if under-reported. The questionnaires used to assess sedentary behaviours in ELSA have not been validated against objective measures, although a recent review concluded that questions focusing on television viewing have the strongest reliability and validity among non-occupational sedentary behaviour questions (Clark et al., 2009). Present analyses are cross-sectional and thus cannot determine whether television viewing contributes to or results from phenotype status. While obesity has been associated prospectively with subsequent sitting time (Ekelund et al., 2008), television viewing also seems a plausible risk factor for obesity. A feedback loop may also be involved with sitting leading to worsened metabolic health/obesity status, leading to further sitting.

## Conclusions

Results of this study of older adults indicate that a common type of leisure-time sedentary behaviour varies across metabolic and obesity phenotypes. However, differences were observed between non-obese groups only, suggesting that healthy obesity is not explained through differences in leisure-time sedentary behaviour.

The following are the supplementary data related to this article

Supplementary Table 1 Mean television viewing time (hours per week) by metabolic health and obesity phenotype in the English Longitudinal Study of Ageing (n = 4931).

Supplementary data to this article can be found online at http://dx.doi.org/10.1016/j.ypmed.2014.01.028.

## Conflict of interest

None of the authors have any conflicts of interest to declare.

## Figures and Tables

**Table 1:** Differences in mean weekly hours of television viewing between metabolic health and obesity phenotypes in the English Longitudinal Study of Ageing (n = 4931).

	Model 1^a^ B (95% CI)	Model 2^b^ B (95% CI)
Metabolically healthy non-obese (n = 1895)	0.0 (*reference*)	0.0 (*reference*)
Metabolically unhealthy non-obese (n = 1602)	6.6 (4.8, 8.5)	4.7 (2.9, 6.5)
Metabolically healthy obese (n = 299)	7.4 (4.1, 10.8)	5.8 (2.5, 9.0)
Metabolically unhealthy obese (n = 1135)	10.6 (8.6, 12.7)	7.8 (5.7, 9.8)
p-trend	< 0.001	< 0.001

Data are from wave 4 (2008/9) of the English Longitudinal Study of Ageing (England, UK). Coefficients represent differences in television viewing time (hours per week) compared with the reference group.

a Adjusted for age and sex.

b Further adjusted for marital status (‘married/cohabiting’; ‘single/never married/widowed/divorced/separated’), occupational class (‘managerial/professional’; ‘intermediate’; ‘routine/manual’), limiting long-standing illness (‘no longstanding illness/has longstanding illness but not limiting’; ‘has limiting longstanding illness’), basic and instrumental activities of daily living (‘no reported issues’; ‘one or more reported issues’), depressive symptoms (8-item Centre of Epidemiological Studies Depression Scale score > 3), smoking status (‘never smoked’; ‘ex-smoker’; ‘current smoker’), alcohol consumption (‘daily’; ‘weekly’; ‘monthly’; ‘rarely/never’), and moderate–vigorous physical activity (‘hardly ever or never’; ‘one to three times per month’; ‘once per week or more than once per week’).
