# Supplementary material for: Metabolically healthy obesity: What is the role of sedentary behaviour?
Source: Prev Med. 2014 May;62(100):35–7. doi: 10.1016/j.ypmed.2014.01.028 (PMC3995089; doi:10.1016/j.ypmed.2014.01.028)
Supplement: Supplementary Table 1 — Mean television viewing time (hours per week) by metabolic health and obesity phenotype in the English Longitudinal Study of Ageing (n = 4931). [file mmc1.pdf]

Supplementary Table 1: Mean television viewing time (hours per week) by metabolic health and obesity phenotype in the English Longitudinal Study of Ageing (n=4931)

	Model 1[a]	Model 2[b]
	B (95% CI)	B (95% CI)
Metabolically healthy non-obese (n=1895)	31.4 (30.1, 32.6)	36.1 (33.4, 38.8)
Metabolically unhealthy non-obese (n=1602)	38.0 (36.6, 39.3)	40.8 (38.2, 43.5)
Metabolically healthy obese (n=299)	38.8 (35.7, 41.9)	41.9 (38.1, 45.7)
Metabolically unhealthy obese (n=1135)	42.0 (40.4, 43.6)	43.9 (41.1, 46.7)
p-trend	<0.001	<0.001

Supplementary Table 1 legend: Data are from wave 4 (2008/9) of the English Longitudinal Study of Ageing (England, UK). Mean television viewing time (hours per week) by metabolic health and obesity phenotype. [a]Adjusted for age, sex. [b]Further adjusted for marital status (‘married/cohabiting’; ‘single/never married/widowed/divorced/separated’), occupational class (‘managerial/professional’; ‘intermediate’; ‘routine/manual’), limiting long-standing illness (‘no longstanding illness/has longstanding illness but not limiting’; ‘has limiting longstanding illness’), basic and instrumental activities of daily living (‘no reported issues’; ‘one or more reported issues’), depressive symptoms (CES-D > 3), smoking status (‘never smoked’; ‘ex-smoker’; ‘current smoker’), alcohol consumption (‘daily’; ‘weekly’, ‘monthly’; ‘rarely/never’), moderate-vigorous physical activity (‘hardly ever or never’; ‘one to three times per month’; ‘once per week or more than once per week’).
